# Antioxidant enzyme Prdx1 inhibits osteoclastogenesis via suppressing ROS and NFATc1 signaling pathways

**DOI:** 10.1002/jcp.31431

**Published:** 2024-09-12

**Authors:** Chao Wang, Gang Wang, Fangming Song, Jinmin Zhao, Qian Liu, Jiake Xu

**Affiliations:** ^1^ The Discipline of Pathology and Laboratory, School of Biomedical Sciences The University of Western Australia Perth Western Australia Australia; ^2^ Faculty of Pharmaceutical Sciences, Shenzhen University of Advanced Technology Chinese Academy of Sciences Shenzhen China; ^3^ Guangxi Key Laboratory of Regenerative Medicine Guangxi Medical University Nanning Guangxi China

**Keywords:** antioxidant, NFATc1, osteoclast, Prdx1, ROS

## Abstract

Bone is a dynamic organ which continuously undergoes remodeling throughout one's lifetime. Cellular production of reactive oxygen species (ROS) is essential for regulating bone homeostasis. Osteoclasts, multinucleated giant cells differentiated from macrophage lineage, are responsible for osteolytic bone conditions which are closely linked to ROS signaling pathways. In this study, an anti‐ROS enzyme, peroxiredoxin 1 (Prdx1) was found to be expressed both in bone marrow macrophages and osteoclasts. Recombinant Prdx1 protein was found to dose‐dependently inhibit ROS production and osteoclast differentiation. Mechanistically, Prdx1 protein also attenuated NFATc1 activation as well as the expression of C‐Fos, V‐ATPase‐d2, Cathepsin K, and Integrin αV. Collectively, Prdx1 is a negative regulator on osteoclast formation via inhibiting RANKL‐mediated ROS activity, thus suggesting its potential application for treating osteoclast related disorders.

## Graphical abstract

Abstract Fig. A proposed scheme for the inhibition effect of Prdx1 on osteoclastogenesis. RANKL‐mediated TRAF6 recruitment results in ROS production, Nrf2 activation and Keap1 degradation, as well as NFATc1 activation and downstream osteoclast specific genes. Our results indicated that Prdx1 could suppress osteoclast formation via attenuating ROS and NFATc1 singalling pathways.

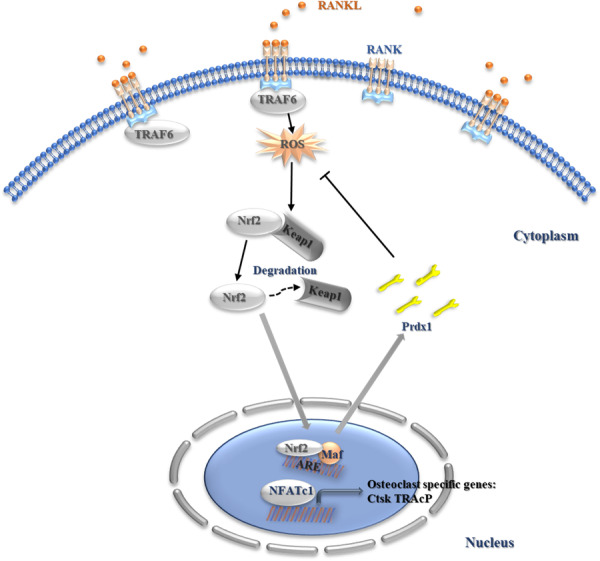

## 1 INTRODUCTION

Osteoclasts and osteoblasts play pivotal roles in governing bone homeostasis, with osteoclasts being the principal cells involved in the degradation of mineral matrix (Teitelbaum, 2000). Skeletal homeostasis is strongly affected by a myriad of factors, such as hormonal factors and aging (Rodan, 1998). During aging, increased osteoclastic bone resorption coupled with reduced osteoblastic bone formation could lead to excessive bone loss (Ferguson et al., 2003). One of the main determinants of this pathological process are reactive oxygen species (ROS) that are mainly produced through mitochondrial electron transport (Nickel et al., 2014). Importantly, oxidative damage caused by ROS is correlated with aging and can lead to tissue degeneration in the long term (Muller et al., 2007). In recent years, several studies have indicated that ROS are critical factors in regulating osteoclast differentiation (Callaway & Jiang, 2015). More importantly, it has been widely reported that ROS are the main factors responsible for bone degeneration and other pathological disorders such as osteoporosis and Paget's disease (Wauquier et al., 2009). While many studies have focused on the aspect of eliminating ROS pharmacologically, increasing evidence now points to the antioxidant enzyme as one of the key components in reducing cellular ROS production (Rajendran et al., 2014). As ROS play an important role in the regulation of osteoclastogenesis (Callaway & Jiang, 2015), identifying novel regulators which can modulate ROS activity in osteoclasts can potentially lead to the development of novel ROS‐targeted treatment of skeletal disorders, such as osteoporosis.

To prevent cellular oxidative stress, cells utilize multiple strategies to eliminate ROS production, including antioxidant enzymatic reaction. A majority of antioxidant enzymes are capable of converting H_2_O_2_ into H_2_O and O_2_ (Kadenbach et al., 2009), with peroxiredoxin (Prdx) family members considered as the most important players in this critical process (Poynton & Hampton, 2014). Prdxs, a ubiquitous family of cystein‐dependent peroxidase enzymes, have emerged as a class of antioxidant enzymes that play a major role in reducing cellular ROS activity by scavenging peroxides and defending against oxidative stress (Perkins et al., 2015). For example, peroxiredoxin 4 (Prdx4), which belongs to one of the peroxiredoxin family members, was found to be secreted by breast and prostate cancer cells and capable of promoting osteoclastogenesis (Rafiei et al., 2015). Although this study points to Prdx4 as a mediator of cancer cell‐induced osteoclast activation, it is currently unclear whether Prdx family members are expressed by osteoclasts and involved in regulating osteoclast activity and function.

It is widely known that a diverse range of transcriptional factors are involved in osteoclast differentiation. For instance, nuclear factor kappa B (NF‐κB) was found to be activated by ROS during an oxidative stress event (Schreck et al., 1991). Furthermore, studies have shown that antioxidants could suppress RANKL‐induced NF‐κB activation in bone marrow macrophages (BMMs) and the bone resorbing function of osteoclasts (Ha et al., 2004). NFATc1, a member of the NFAT transcription factor family, is also known to be a critical component in the RANKL‐induced signaling pathway in osteoclasts. First identified in T‐cells, NFATc1 was reported to induce an auto‐amplification in response to its own promoter activation, leading to a robust induction of osteoclast activation (Asagiri et al., 2005). In addition, there was strong evidence that ROS are essential in inducing NFATc1 amplification and NFATc1‐related gene expression in osteoclasts (Kim et al., 2010).

In this study, we found that antioxidant enzyme Prdx1 is expressed in osteoclasts and is involved in regulating osteoclast differentiation by modulating ROS. Furthermore, we investigated the intracellular mechanisms underlying the suppressive effect of Prdx1 on RANKL‐mediated osteoclastogenesis, pointing to its potential application for the treatment of osteoclast related disorders.

## 2 MATERIALS AND METHODS

### 2.1 Reagents

Peroxiredoxin 1 (Prdx1) recombinant protein was purchased from Abnova (Australia). Anti‐NFATc1, Anti‐c‐fos, Anti V‐ATPase‐d2 and Anti‐Ctsk antibodies were purchased from Santa Cruz. Anti Integrin αv antibody was purchased from Sigma‐Aldrich. Anti‐goat immunoglobulin G (IgG) Peroxidase Conjugate and Anti‐mouse IgG Peroxidase Conjugate were purchased from Sigma‐Aldrich. Anti‐mouse β‐actin (JLA‐20) antibody was purchased from Calbiochem. RANKL and M‐CSF were used as previsouly described (Xu et al., 2000). BMP2 recombinant proteins were obtained from R&D Systems Inc. 6‐Carboxy‐2′,7′‐dichlorodihydrofluorescein diacetate was purchased from Thermo Fisher Scientific. Luciferase assay system was purchased from Promega Corporation. The specific mouse primers used in quantitative PCR (qPCR) reactions were designed as follows: mouse TRAcP (Acp5) (forward: 5′‐TGTGGCCATCTTTATGCT‐3′; reverse: 5′‐GTCATTTCTTTGGGGTT‐3′), cathepsin K (Ctsk) (forward: 5′‐GGGAGAAAAACCTGAAGC ‐3′; reverse: 5′‐ATTCTGGGGACTCAGAGC‐3′), Peroxiredoxin‐1 (Prdx1) (forward: 5′‐TGGCTCGACCCTGCTGATAG‐3′; reverse: 5′‐GCAATGATCTCCGTGGGACA‐3′), housekeeping gene Hprt (forward: 5′‐CAGTCCCAGCGTCGTGATTA‐3′; reverse: 5′‐TGGCCTCCCATCTCCTTCAT‐3′). All primers were purchased from Sigma‐Aldrich.

### 2.2 BMMs isolation and osteoclast culture

BMMs were isolated from the femurs and tibias of 12 weeks C57BL/6 mice and cultured in complete α‐MEM with 10 ng/mL M‐CSF. Cells were seeded either in six‐well plate at the concentration of 8 × 10^4^ cells/well or in 96‐well plate at the density of 5 × 10^3^ cells/well. Culture medium was changed (complete medium with 10ng/mL M‐CSF and 50 ng/mL RANKL) after the cells were adherent. Osteoclastogenesis was observed after two times of changing medium and RANKL stimulation. TRAcP staining or total RNA extraction were performed.

### 2.3 TRAcP staining and hydroxyapatite resorption assay

BMMs were extracted from 12 weeks female C57BL/6 mice and cultured in 96 well plate to generate osteoclasts. After the osteoclasts were formed, cells were washed by 1x phosphate‐buffered saline for one time, and fixed by 2.5% glutaraldehyde (v/v). The cells were then stained by TRAcP staining solution and TRAcP positive multinucleated cells with three or more nuclei were scored as osteoclast‐like (OCL) cells. To perform hydroxyapatite resorption assay, BMMs were seeded and cultured with complete medium, 10ng/mL M‐CSF and 50 ng/mL RANKL in six‐well collagen coated plates. After 4 days RANKL stimulation, preosteoclasts were generated and observed under microscopy. Cells were then detached gently using cell dissociation solution and seeded into 96‐well hydroxyapatite plate at 5 × 10^3^ cells/well. Preosteoclasts were incubated in RANKL and M‐CSF contained medium to form mature osteoclasts. After 48 h, half of the wells were performed TRAcP staining for calculating OCL cells in each well. Cells in the remaining wells were bleached and discarded, resorbed areas were photographed by microscopy and percentages of hydroxyapatite coat resorbed by osteoclasts were quantified by ImageJ software (NIH, Bethesda, MD).

### 2.4 RNA isolation and complementary DNA (cDNA) synthesis

Total RNA was extracted from osteoclasts by Trizol according to manufacturer's instructions. RNA concentration was measured using Biophotometer Plus (Eppendorf). To synthesis cDNA, RNA samples were first mixed with Oligo dT, nuclease‐free water. For one reaction, 1 μg RNA diluted in 15 μL nuclease‐free water, 0.25 μL Oligo dT (100 μM) and 2.5 μL nuclease‐free water were mixed together and the total volume is 17.75 μL. The mixed sample was heated at 75°C for 3 min in Eppendorf thermocycler. The mixture was incubated at 42°C for 1 h and heated up to 92°C for 10 min and 4°C indefinitely inside Eppendorf thermocycler to end the reaction. All cDNA samples were kept at −20°C for storage.

### 2.5 Quantitative PCR

cDNAs were isolated as previously described, followed by dilution (1/5) in nuclease‐free water. The following cycling parameters for qPCR reaction were used: 50°C for 120 s (holding stage), 95°C for 10 min; 40 cycles at 95°C for 15 s (denaturation), and 60°C for 60 s (primer annealing); followed by 95°C for 15 s, 60°C for 60 s, and 95°C for 15 s to measure the melt curve of PCR reaction products. Each gene was normalized with a house‐keeping gene, and the analysis was then carried out using the Livak relative gene quantification method (Livak & Schmittgen, 2001).

### 2.6 ROS measurement assay

Intracellular ROS activities were investigated using 6‐carboxy‐2′,7′‐dichlorodihydrofluorescein diacetate (carboxy‐H_2_DCFDA) dye according to the manufacturer's protocol. Briefly, BMMs were seeded in a black 96‐well plate and incubated with Prdx1 for 24 h. Following incubation, RANKL (50 ng/mL) was added to the RANKL(+) and RANKL(+) + Prdx1 groups. The cells were then starved and stained with carboxy‐H_2_DCFDA, and fluorescence intensity changes were measured over 20 min using a microplate reader (BMG) at extraction wavelength of 485 nm and emission wavelength of 535 nm to assess intracellular ROS activity. To further evaluate the effect of Prdx1 on RANKL‐induced ROS activity in preosteoclasts, BMMs were seeded in 35 mm glass‐bottom micro‐well dishes and pretreated as described. After 24 h, RANKL was added to the RANKL(+) and RANKL(+) + Prdx1 groups, and cells were incubated for an additional 24 h before being stained with carboxy‐H_2_DCFDA. The dishes were then transferred to the live cell chamber (37°C and 5% CO_2_) of an inverted A1Si confocal microscope (Nikon), where live cell images were captured, and fluorescence intensities were measured to assess ROS activity. Images of living BMMs with different treatments were captured by confocal microscope to show the effect of RANKL‐induced ROS activity as reported (Wang et al., 2020).

### 2.7 Luciferase reporter assay

RAW_264.7_ cells transfected with ARE or NFAT luciferase reporter genes were seeded into 48‐well plate at a density of 1.5 × 10^5^ and 5 × 10^4^ cells, respectively, as reported (Qiu et al., 2021, 2023). Following cell attachment to the bottom of the wells, cells were pretreated with Prdx1 for 1 h, followed by 24 h of stimulation with 50 ng/mL RANKL. Culture medium was discarded and cells were then lysed with Luciferase Lysis Buffer. The cell lysates were centrifuged at 14,000 rpm at 4°C for 20 min, and supernatants were transferred to new 1 mL tubes. A total of 50 μL of the supernatants were transferred to a 96‐well plate and luciferase activity was measured using the Promega luciferase kit and a BMG POLARstar Optima luminescence reader (Germany).

### 2.8 Western blot assay

BMMs were cultured in six‐well plates and subjected to different treatments as indicated by the respective experiments. The concentration of proteins in the samples was determined using the Bio‐Rad protein assay. The sodium dodecyl sulfate–polyacrylamide gel electrophoresis was used to separate proteins based on their molecular weight. The nitrocellulose membrane was incubated with primary antibodies overnight at 4°C. A peroxidase‐conjugated IgG secondary antibody was added and incubated. The membrane was washed and protein detection was carried out using the Western Lightening Ultra Extra Sensitivity kit. The ImageQuant LAS‐4000 (NJ, USA) was used for detection, and ImageJ software used for band intensity analysis.

### 2.9 Bone nodule formation assay

Preosteoblasts were obtained from calvaria of neonatal mice and cultured with dexamethasone (5 nM), β‐glycerophosphate (10 mM) and ascorbate (50 μg/mL) with the addition of Prdx1 (200 ng/mL) or not. BMP2 (25 ng/mL) was used as a positive control. After 21 days, cells was fixed and stained with Alizarin red S (ARS). Relative mineralized areas were calculated and quantified by ImageJ.

## 3 RESULTS

### 3.1 Prdx1 suppressed RANKL‐induced osteoclastogenesis but had little effect on osteoclastic bone resorption

**Figure 1:**
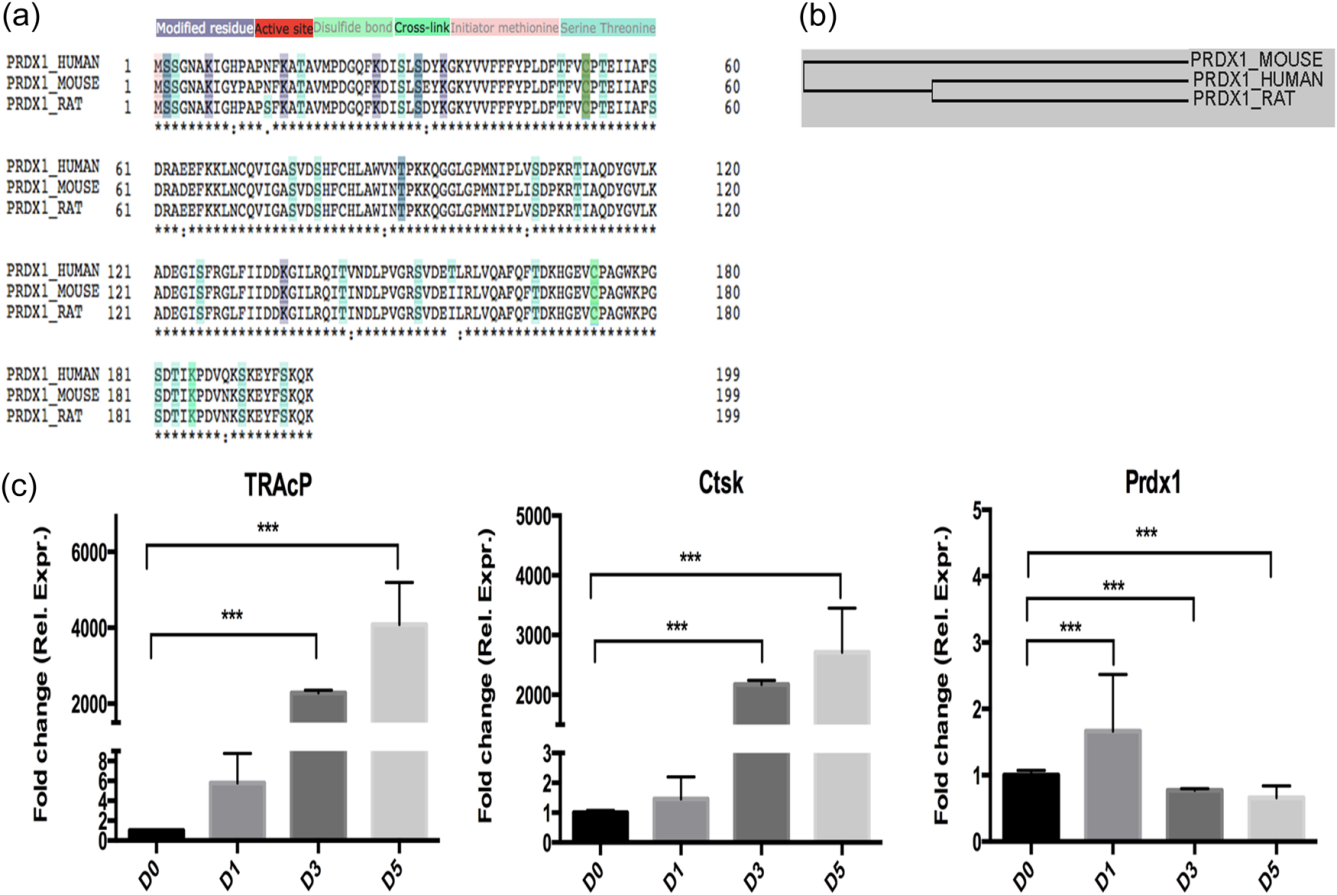
Prdx1 and osteoclast specific gene expressions during osteoclast formation. (a) Prdx1 sequence alignment for human, mouse and rat. (b) Cluster analysis of Prdx1 in human, mouse and rat using online Uniprot software. (c) q‐PCR amplification of Prdx1 and osteoclast specific genes TRAcP (Acp5) and Ctsk during osteoclastogenesis. Data represent the mean ± SEM; *p < 0.05, **p < 0.01, and ***p < 0.001; *n* = 3.

To analyze the bioinformatic features of Prdx1, sequences alignments for human, mouse and rat were carried out. The results showed that the amino acid sequences of mouse, human and rat shared 94.975% similarity, and human Prdx1 is more related to rat followed by mouse (Figure 1a,b). To further examine the expression of Prdx1 gene during osteoclast differentiation, qPCR was performed. Compared to the highly upregulated osteoclast marker genes such as TRAcP (Acp5) and Ctsk, the expression of Prdx1 was significantly increased at the early stage of osteoclast differentiation (Figure 1c).

**Figure 2:**
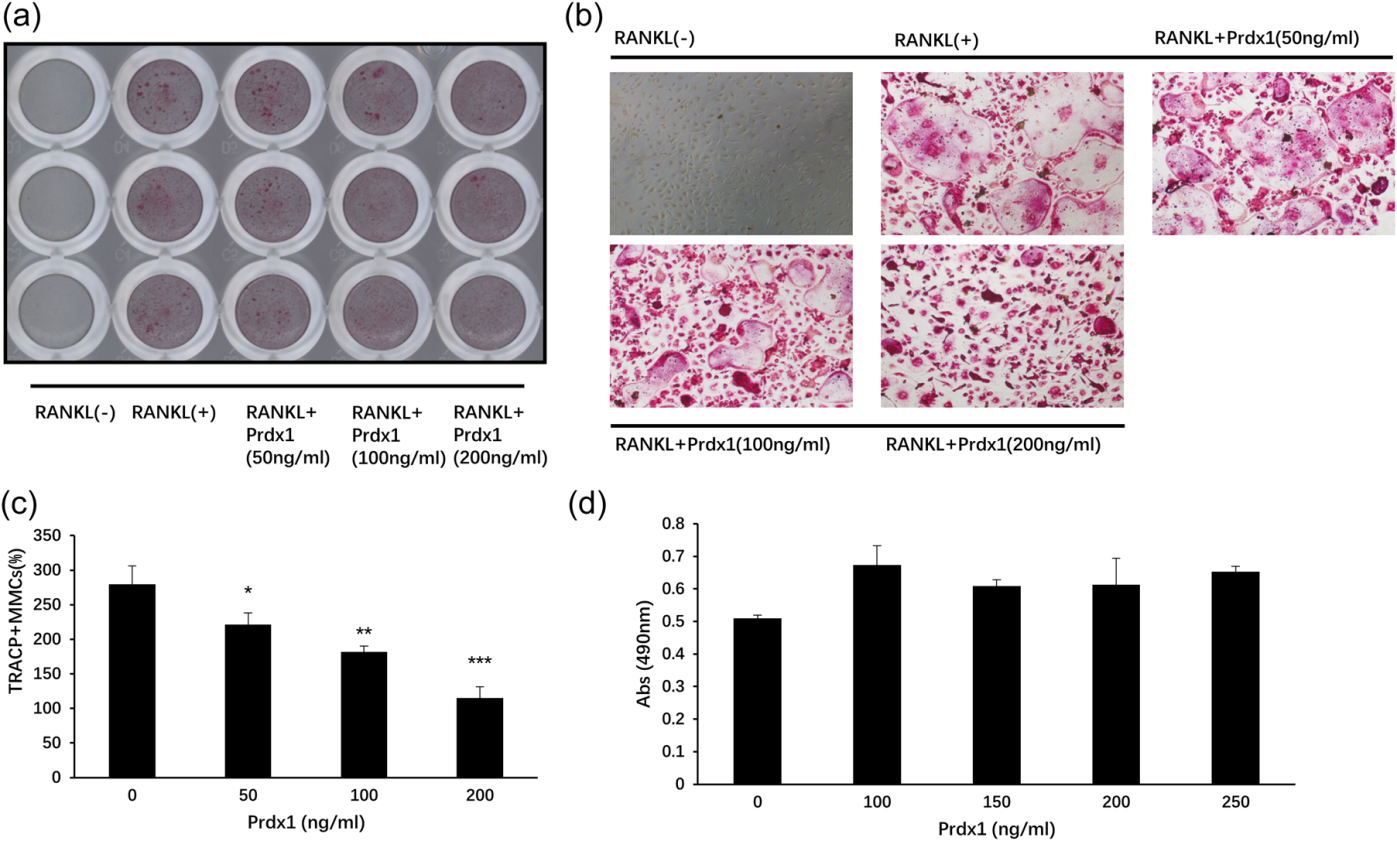
Prdx1 inhibited osteoclast differentiation. (a) BMMs isolated from C57BL/6 mice and cultured with 10 ng/mL M‐CSF and 50 ng/mL RANKL for indicated days. TRAcP staining was then performed to observe the osteoclasts. (b) Random fields of stained osteoclasts in 96‐well plate were selected and images were taken under Nikon inverted microscopy. (c) TRAcP positive multinucleated cells with three or more nuclei were scored as osteoclast‐like (OCL) cells. (d) BMMs were collected from C57BL/6 mice and treated with 10 ng/mL M‐CSF and different concentrations of Prdx1. Cell proliferation was measured by an MTS assay after 48 h incubation. Data represent the mean ± SEM; *p < 0.05, **p < 0.01, and ***p < 0.001; *n* = 3. BMMs, bone marrow macrophages.

**Figure 3:**
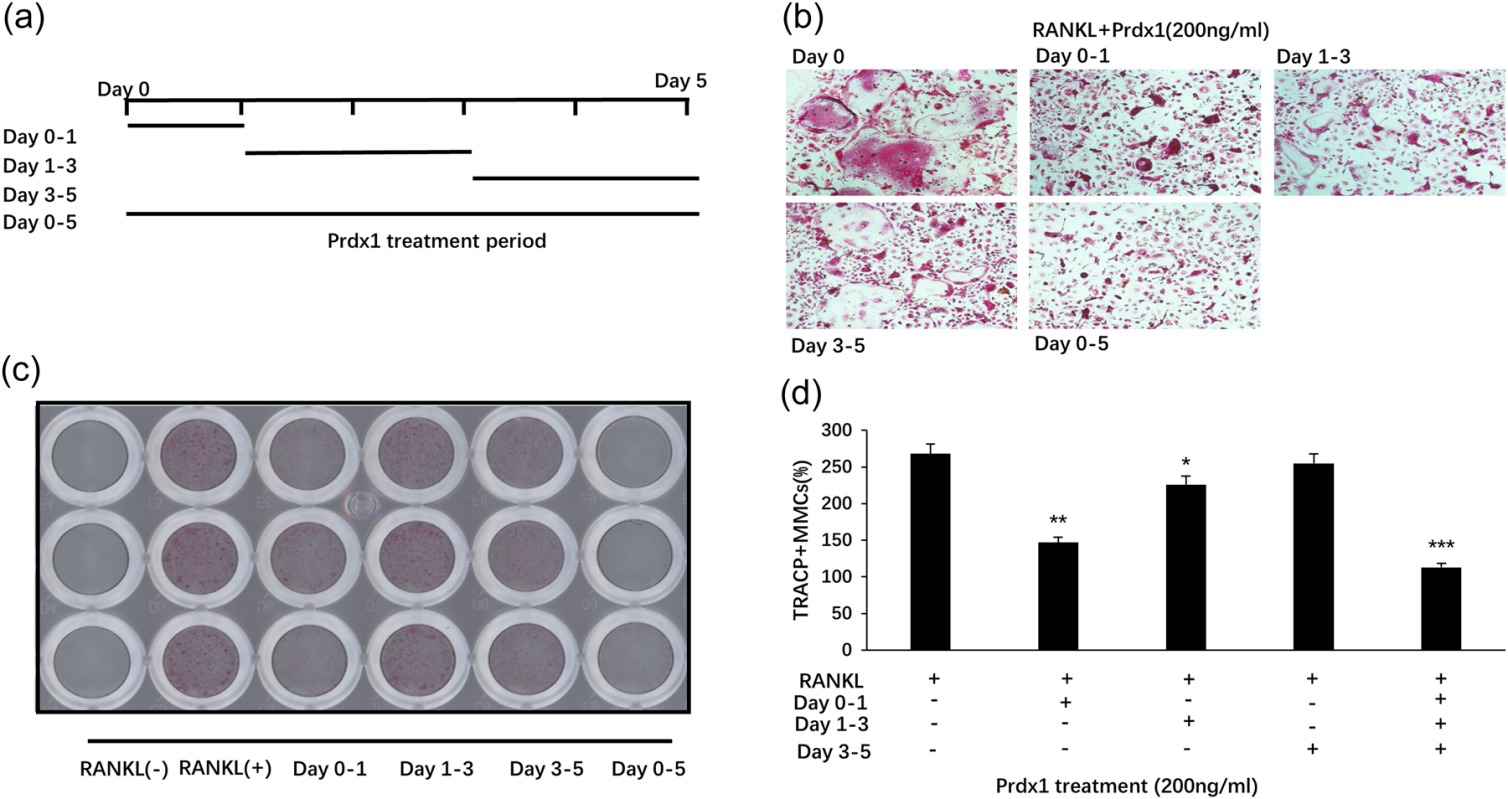
Prdx1 suppressed the early stage of osteoclastogenesis induced by RANKL. (a) BMMs were cultured with M‐CSF, and 50 ng/mL RANKL and 200 ng/mL Prdx1 were added at certain time points. The treatment period was shown as the diagram. (b) TRAcP staining was then subjected to observe the osteoclasts. (c) Random fields of TRAcP positive image were taken for each experimental date. (d) The OCL number was counted and analyzed. Data represent the mean ± SEM; *p < 0.05, **p < 0.01, and ***p < 0.001; *n* = 3. BMMs, bone marrow macrophages; OCL, osteoclast‐like.

**Figure 4:**
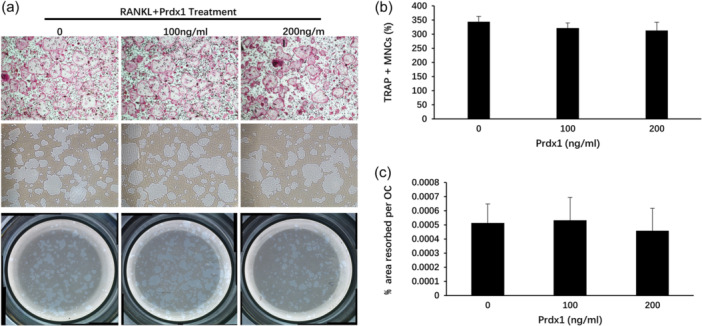
Prdx1 had little impact on hydroxyapatite resorption. (a) BMMs were seeded in six‐well collagen‐coated plate and treated with 10 ng/mL M‐CSF and 50 ng/mL RANKL. After mature osteoclasts were formed, cells were gently detached and seeded into 96‐well hydroxyapatite plate. Mature osteoclasts were treated with 50 ng/mL RANKL and different concentrations of Prdx1 for 48 h. Half of the cells in each experimental group were stained by TRAcP to calculate the OCL number, the remaining groups of cells were bleached to measure the resorption area. (b) The OCL number was counted, and (c) the percentage of hydroxyapatite resorbed area was measured by ImageJ software. *n* = 3. BMMs, bone marrow macrophages; OCL, osteoclast‐like.

To investigate the biological function of Prdx1 in osteoclast formation, BMMs were stimulated by RANKL and M‐CSF in the presence of recombinant Prdx1 protein to form osteoclasts. Increasing concentrations of Prdx1 from 50 to 200 ng/mL inhibited TRAcP‐positive osteoclast formation (Figure 2a–c). To confirm that Prdx1 had no toxicity effect on cells, a cell proliferation assay was performed. As shown in Figure 2d, Prdx1 had no cytotoxic to BMMs. Considering the time‐dependent expression of Prdx1 at the early stage of osteoclast differentiation, a time‐course osteoclastogenesis assay was also conducted. Prdx1 treatment was applied at specific time points during osteoclastogenesis (Figure 3a). The results showed that Prdx1 inhibited RANKL‐induced osteoclastogenesis at the early stage of osteoclastogenesis from days 0 to 1 (Figure 3b–d). To determine the effect of Prdx1 on osteoclastic function, hydroxyapatite resorption assay was performed. Resorbed areas were observed both in the absence or presence of Prdx1 treatments with no significant changes between groups (Figure 4a–c). These results suggested that Prdx1 had little impact on osteoclastic resorption activity.

### 3.2 Prdx1 suppressed intracellular ROS production during the early stages of osteoclastogenesis

**Figure 5:**
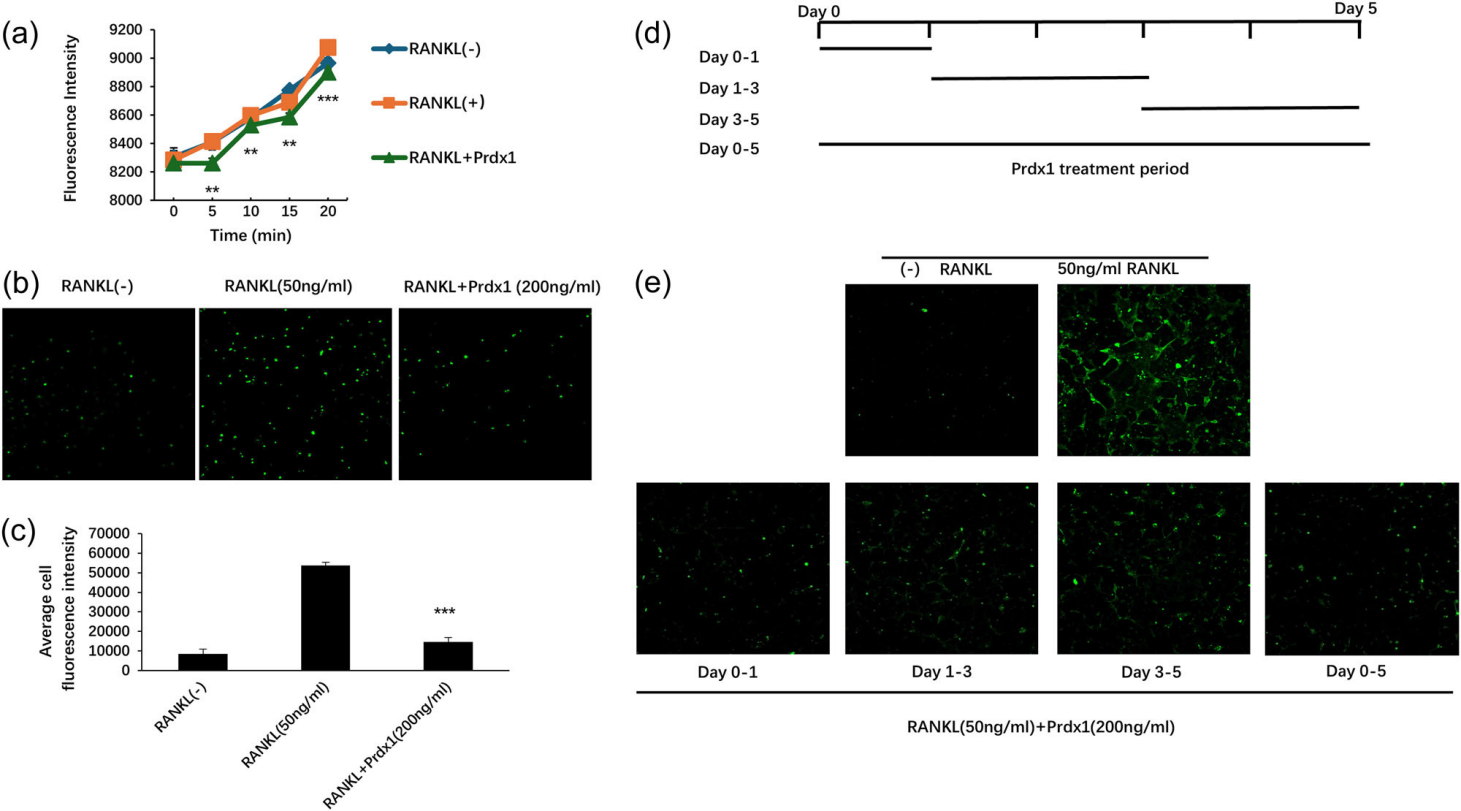
Prdx1 suppressed intracellular ROS production in the early stage of osteoclastogenesis. (a) BMMs were seeded in black 96‐well plate contained 10 ng/mL M‐CSF culture medium. Cells were treated with or without RANKL (50 ng/mL) and Prdx1 (200 ng/mL), respectively, for 24 h after. ROS level was determined by 6‐carboxy‐2′,7′‐dichlorodihydrofluorescein diacetate (carboxy‐H_2_DCFDA) dye using a BMG fluorescent plate reader. (b) BMMs were seeded into 35 mm glass bottom micro‐well dishes, and preosteoclast ROS activity for the described treatment groups was detected by inverted A1Si confocal microscope after 24 h. (c) Fluorescence intensity was calculated using NIS Elements imaging software. (d) BMMs were cultured in 35 mm glass bottom micro‐well dishes with M‐CSF (10 ng/mL) and stimulated by RANKL (50 ng/mL), Prdx1 treatment was performed in specific time points. (e) Carboxy‐H_2_DCFDA dye was used to mark the ROS and fluorescent was captured by inverted A1Si confocal microscope. Data represent the mean ± SEM; *p < 0.05, **p < 0.01, and ***p < 0.001; *n* = 3. BMMs, bone marrow macrophages; ROS, reactive oxygen species.

To determine the antioxidative effects of Prdx1 on osteoclasts, we measured the ROS production of preosteoclasts following the treatment with Prdx1. As shown in Figure 5a, RANKL stimulation led to a significant increase in ROS activity within 20 min at the initial stage. However, pretreatment with Prdx1 markedly inhibited this RANKL‐induced ROS upregulation. To further evaluate the effect of Prdx1 on RANKL‐induced ROS activity in preosteoclasts, BMMs were seeded in 35 mm glass‐bottom micro‐well dishes and pretreated as described. Considering the sensitivity of ROS to environmental factors such as light, oxygen, and temperature, we ensured a stable environment to accurately assess the influence of Prdx1 on RANKL‐induced ROS activity at the preosteoclast stage. In the subsequent experiment (Figure 5b,c), following 24 h of RANKL stimulation, Prdx1 significantly reduced the RANKL‐induced ROS levels, as indicated by the measured fluorescence intensities. To investigate the temporal effects of Prdx1 during osteoclastogenesis, Prdx1 was administered at various time points throughout the process (Figure 5d). As shown in Figure 5e, RANKL‐induced ROS activity was significantly elevated during osteoclastogenesis. However, treatment with Prdx1 effectively inhibited ROS activity at the early stages (days 0–1) of osteoclast differentiation, while showing minimal suppression at the later stages (days 3–5).

### 3.3 Prdx1 inhibited RANKL‐induced ARE transcriptional activation and osteoclast marker gene expressions

**Figure 6:**
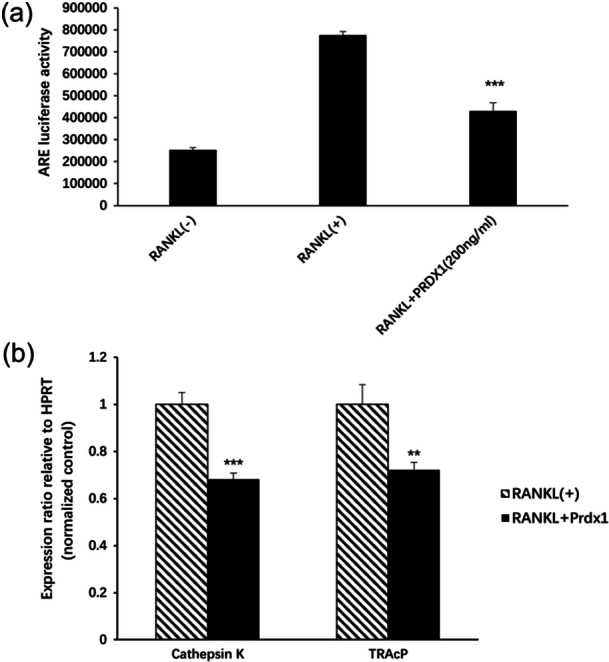
Prdx1 inhibited RANKL‐induced ARE transcription activation and osteoclast marker gene expression. (a) RAW_264.7_ cells with stably transfected ARE luciferase reporter gene were pretreated with or without Prdx1 for 1 h and stimulated with 50 ng/mL RANKL. ARE activity was utilized by Luciferase reporter assay using BMG POLARstar Optima luminescence reader. (b) BMMs were extracted from C57BL/6 mice, cultured with 10 ng/mL M‐CSF and 50 ng/mL RANKL and treated or untreated with 200 ng/mL Prdx1 for 5 days. Total RNA was extracted, and q‐PCR was used to identify the expressions of osteoclast specific genes encoding Cathepsin K and TRAcP proteins. Data represent the mean ± SEM; *p < 0.05, **p < 0.01, and ***p < 0.001 compared with RANKL‐untreated control groups; *n* = 3. q‐PCR, quantitative PCR.

The antioxidant response element (ARE) luciferase reporter was used to determine the transcription factor nuclear factor erythroid 2‐related factor 2 (Nrf2). To examine the role of Prdx1 in RANKL‐induced ARE transcription activity, ARE activity was measured by a luciferase reporter assay using BMG POLARstar Optima luminescence reader. As shown in Figure 6a, RANKL‐induced ARE transcription activity was repressed by Prdx1. In addition, qPCR was performed to examine the expression of osteoclast specific genes encoding Cathepsin K (Ctsk) and TRAcP proteins. As indicated in Figure 6b, the expressions of Cathepsin K and TRAcP (Acp5) encoding genes were significantly reduced by Prdx1 treatment.

### 3.4 Prdx1 inhibited RANKL‐induced NFATc1 signaling pathways and osteoclast specific proteins expression

**Figure 7:**
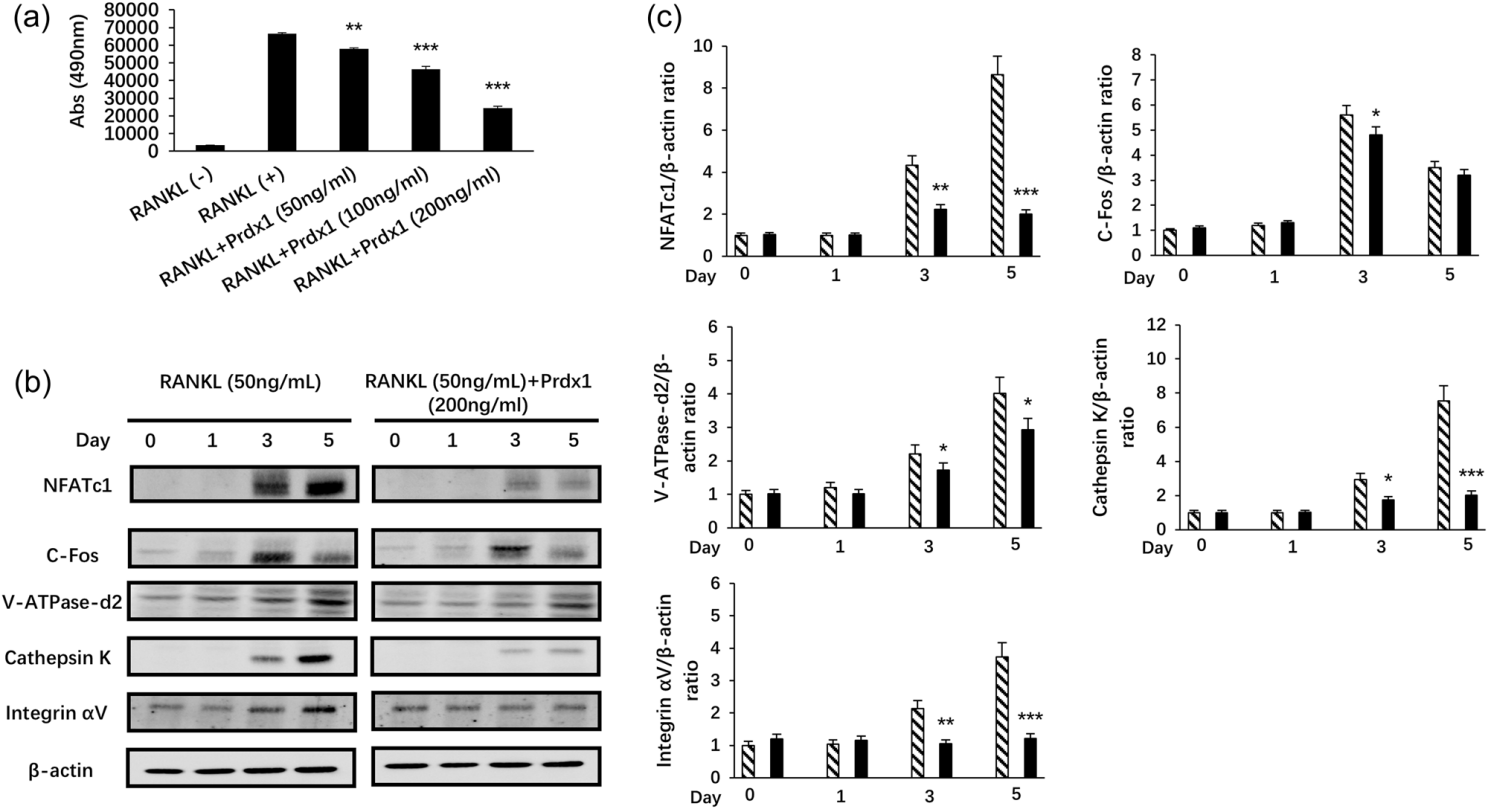
Prdx1 inhibited RANKL‐induced NFATc1 signaling pathways and osteoclast specific protein expression. (a) RAW_264.7_ cells transfected with NFATc1 reporter gene were pretreated with or without certain doses of Prdx1 for 1 h followed by RANKL stimulation for 24 h. Luciferase reporter assay was carried out to measure NFAT activity. (b) Representative Western Blot assay image showing the proteins expression level of NFATc1, C‐Fos, V‐ATPase‐d2, Cathepsin K and Integrin αV. BMMs were isolated from C57BL/6 mice, cultured with 10 ng/mL M‐CSF and 50 ng/mL RANKL and treated or untreated with 200 ng/mL Prdx1 for 0, 1, 3, and 5 days before protein collection. (c) Quantitative analysis of band intensity ratios were calculated by ImageJ. Data represent the mean ± SEM; *p < 0.05, **p < 0.01, and ***p < 0.001 compared with RANKL‐untreated control groups; *n* = 3. BMMs, bone marrow macrophages.

To evaluate NFATc1 transcription activation, RAW_264.7_ cells stably transfected with an NFATc1 luciferase reporter construct were used. We found that RANKL‐induced NFATc1 activation was significantly inhibited by Prdx1 in a dose dependent manner (Figure 7a). To further investigate the protein expression level of NFATc1, Western Blot assay was performed. BMMs were isolated from C57BL/6 mice, cultured with 10 ng/mL M‐CSF and 50 ng/mL RANKL and stimulated with or without 200 ng/mL Prdx1 for 0, 1, 3 and 5 days before protein collection. NFATc1 protein expression was found to be suppressed by Prdx1 treatment (Figure 7b). Consistently, protein expression levels of c‐Fos, V‐ATPase‐d2, Cathepsin K and Integrin αV were also inhibited (Figure 7c). The results demonstrated that Prdx1 effectively inhibited the upregulation of c‐Fos, V‐ATPase‐d2, Cathepsin K, and Integrin αV induced by RANKL.

### 3.5 Osteoblast activity was not affected by Prdx1

**Figure 8:**
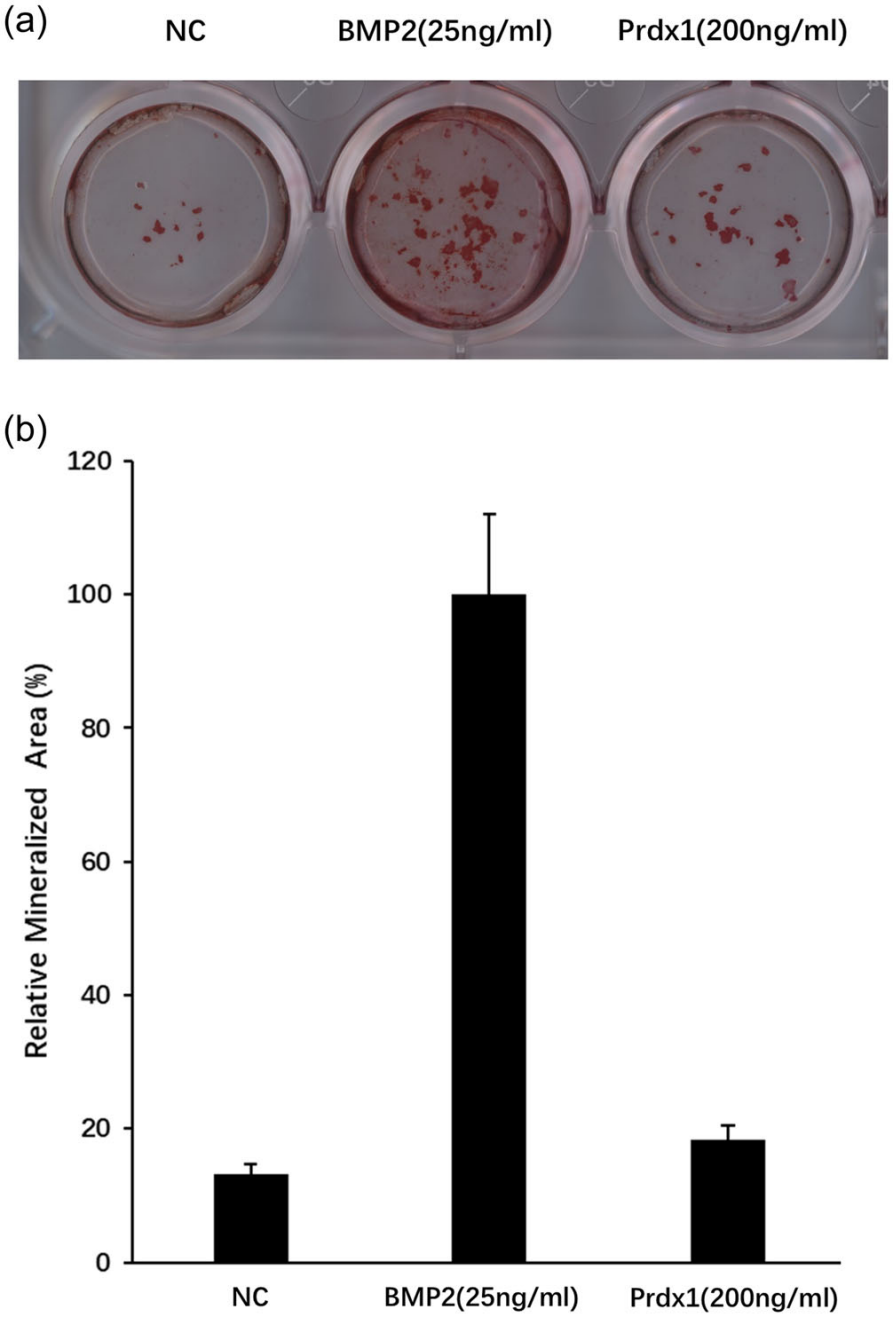
Bone nodule formation was not affected by Prdx1. Preosteoblasts were isolated from calvaria of neonatal mice and cultured with dexamethasone, β‐glycerophosphate and ascorbate. (a) After 21 days, cells was stained by Alizarin red S (ARS). (b) Relative mineralized areas were calculated and quantified by ImageJ software. Data are presented as mean/fold change ± SEM. NS = non significant. *n* = 3.

To gain insights into the potential impact of Prdx1 on osteoblast activity, the formation of bone nodules was assessed. Preosteoblasts were isolated from calvaria of neonatal mice and cultured with dexamethasone (5 nM), β‐glycerophosphate (10 mM) and ascorbate (50 µg/mL) with the addition of Prdx1 or not. BMP2 was used as a positive control. After 7 days, cells were stained by Alizarin red S and relative mineralized areas were quantified. The results showed that addition of Prdx1 has little or limited effect on bone nodule formation (Figure 8).

## 4 DISCUSSION

Bone tissue is constantly remodeled to maintain skeletal homeostasis throughout the lifespan. This biological process is tightly regulated by two main cell types: osteoclasts and osteoblasts. Osteoclasts, which are giant multinucleated cells formed from the macrophage lineage, are responsible for resorbing bone and releasing mineral matrix (Charles & Aliprantis, 2014). In contrast, osteoblasts are differentiated from mesenchymal stem cells and they play a major role in bone formation (Deschaseaux et al., 2009). The delicate balance between the resorption and formation of bone tissues is essential for healthy skeletal growth and maintenance (Kular et al., 2012). However, as aging progresses, increased osteoclastic activity leads to the deterioration of bone structures, mass and integrity. Throughout the process of bone remodeling, osteoclasts play a key role in the resorption of old bone matrix and mediates new bone synthesis via crosstalk with osteoblasts, thus maintaining the homeostatic balance in the bone (Boyle et al., 2003). Interestingly, there is ample evidence showing that ROS, the main contributors to oxidative stress (Muller et al., 2007), are closely associated with aging and the differentiation of osteoclasts (Almeida, 2012). Moreover, previous studies have shown that ROS are capable of promoting osteoclast formation in vitro (Suda et al., 1993). One of the key mechanisms of osteoclast function involves ROS production, which is activated by RANKL in vitro (Chen et al., 2019; Yip et al., 2005). Importantly, ROS were found to be involved in cellular oxidative stress and the loss of bone mass in vivo (Callaway & Jiang, 2015). To combat with oxidative stress, cells employ antioxidant enzymes to remove ROS production (Rajendran et al., 2014). Therefore, understanding the roles of antioxidant enzymes (such as the Prdx family members) in the regulation of osteoclasts could further enhance our understanding of osteoclast biology and may have direct implications for a future treatment of skeletal disorders, such as osteoporosis.

ROS are known to be potent stimulators of osteoclastic bone resorption, a primary cause for the pathophysiology of bone destruction (Bax et al., 1992). Therefore, targeting the production of ROS or reducing intracellular ROS level in the osteoclasts may prove to have promising therapeutic implications. In the current study, we focused on Prdx1, an antioxidant enzyme that is capable of eliminating intracellular ROS (Rhee et al., 2012). Importantly, ROS are associated with NFATc1 activation, a key transcription factor that regulates osteoclast differentiation (Boyle et al., 2003). For instance, it has been reported that the suppression of ROS production can lead to the attenuation of NFATc1 induction (Han et al., 2007). Ctsk has been described as a major bone degrading enzyme that regulates bone matrix resorption by its unique function of cleaving type I collagen (Nallaseth et al., 2013; Wilson et al., 2009). TRAcP is widely acknowledged as an osteoclast biomarker that plays a pivotal role in bone development (Hayman, 2008). Consistently, we have found that Prdx1 inhibited the gene expression of Ctsk and TRAcP.

Antioxidant enzymes primarily target ROS and decompose hydrogen peroxide into water and oxygen (Day, 2014). The Prdx family members have been demonstrated as crucial antioxidant enzymes that play an important role in degrading hydroperoxides to water, thus protecting the cells against oxidative stress and ROS‐induced cell damage (Poynton & Hampton, 2014). More recently, it has been reported that a Prdx family member, Prdx4, can regulate cancer‐induced osteoclast activation (Rafiei et al., 2015). This suggests that Prdx family members may be crucial in regulating osteoclast differentiation, a novel idea which formed the basis of our current study. While we examined the ROS and NFATc1 signaling mechanisms of Prdx1 in osteoclasts in the current study, it is highly possible that Prdx1 may also regulate other osteoclast signaling pathways, such as JNK and p38 mitogen‐activated protein kinase (MAPK) pathways (Li et al., 2014). Indeed, Prdx1 is known to possess anticancer properties and has been described as a pivotal factor which regulates JNK and p38 MAPK pathways, thus protecting the cells against MAPK/p38‐induced cell apoptosis (Ding et al., 2017). ROS production induced by RANKL could lead to activation of TRAF6, which in return, results in Keap1 degradation and upregulation of downstream target genes.

Published studies underscore the crucial role of Prdx1 in protecting Keap1 from RANKL‐induced degradation in osteoclast progenitor cells by mitigating oxidative stress. Prdx1, a key antioxidant enzyme, functions by directly scavenging ROS such as hydrogen peroxide, thereby preventing oxidative damage to vital cellular components, including proteins like Keap1 (Ishii et al., 2012; Rhee, 2016). The process of osteoclast differentiation, driven by RANKL stimulation, is associated with a significant increase in intracellular ROS levels. This rise in ROS can lead to the oxidative modification of Keap1, a critical regulatory protein in the Nrf2 pathway, which is essential for cellular antioxidant defense (Kobayashi et al., 2004; Lau et al., 2008). Under oxidative stress, the cysteine residues in Keap1 become oxidized, triggering its ubiquitination and subsequent proteasomal degradation. This degradation process releases Nrf2, allowing it to translocate into the nucleus and activate the expression of genes involved in antioxidant responses (Kobayashi et al., 2004; Lau et al., 2008). However, research has shown that Prdx1 can intervene in this pathway by inhibiting the ROS‐induced degradation of proteins, including signaling molecules crucial for cellular homeostasis. By reducing the levels of ROS, Prdx1 could preserve Keap1, thus preventing its degradation and modulating the activation of the Nrf2 pathway (Kanzaki et al., 2013). Through modulating the extent of oxidative stress and the subsequent Nrf2 activation, Prdx1 potentially influences the differentiation of osteoclasts. These findings suggest that Prdx1 is not only essential for protecting cells from oxidative damage but also for maintaining the stability of critical signaling pathways during osteoclastogenesis.

It was revealed that Prdx1 significantly inhibits RANKL‐induced osteoclast formation in a dose‐dependent manner and during the early stages of osteoclastogenesis. Hydroxyapatite resorption assay demonstrated that Prdx1 does not affect osteoclastic resorption, indicating that the primary regulatory effect of Prdx1 is on osteoclast differentiation rather than function. Further investigations on the mechanisms revealed that Prdx1 affects osteoclast differentiation through the regulation of ROS. More specifically, Prdx1 was found to suppress intracellular ROS production during early stage of osteoclastogenesis. Furthermore, RANKL‐induced ARE transcriptional activation was inhibited by Prdx1. These data are in line with the general role of Prdx1 as an antioxidant enzyme that plays a key role in eliminating ROS. ROS activity is known to be generated by RANKL‐induced stimulation which also induces Ca^2+^ oscillation, leading to the upregulation and auto‐amplification of NFATc1 (Kim et al., 2010). In our current studies, we demonstrated that NFATc1 activation is suppressed by Prdx1 both in transcriptional activity and protein expression. The results demonstrated that Prdx1 effectively inhibits the upregulation of C‐Fos, V‐ATPase‐d2, Cathepsin K, and Integrin αV.

In conclusion, this study demonstrates that Prdx1 plays a critical role in the regulation of osteoclastogenesis by mediating ROS activity. Furthermore, Prdx1 could directly regulate NFATc1 pathways and its downstream targets in osteoclasts. These data suggest that Prdx1 may serve as a therapeutic agent for the treatment of osteoclast related conditions such as osteolysis and osteoporosis.

## AUTHOR CONTRIBUTIONS

Chao Wang carried out most of the experiments and data analysis. Gang Wang, Fangming Song, and Qian Liu provided experimental assistance, design and evaluations. Jiake Xu, Jinmin Zhao and Chao Wang participated in drafting manuscript. Jiake Xu supervised the project, coordinated the studies and approved the final manuscript.

## CONFLICT OF INTEREST STATEMENT

The authors declare no conflicts of interest.
